# Mechanistic Aspects of Condensed- and Gaseous-Phase Activities of Some Phosphorus-Containing Fire Retardants

**DOI:** 10.3390/polym12081801

**Published:** 2020-08-11

**Authors:** Ananya Thomas, Malavika Arun, Khalid Moinuddin, Paul Joseph

**Affiliations:** Institute for Sustainable Industries and Liveable Cities, Victoria University, P.O. Box 14428, Melbourne, VIC 8001, Australia; malavika.arun@live.vu.edu.au (M.A.); khalid.moinuddin@vu.edu.au (K.M.); paul.joseph@vu.edu.au (P.J.)

**Keywords:** passive fire protection, phosphorus compounds, char analyses, mechanisms of flame retardance

## Abstract

As a part of our ongoing investigations on passively fire protecting polymeric materials, we have been employing both reactive and additive routes involving phosphorus-containing compounds. These included inorganic and organic substances, and in the latter case, the phosphorus-bearing groups differed in terms of the chemical environments (phosphite, phosphate, phosphine, phosphine oxide and phosphonate ester) and oxidation state of the P atom (i.e., III, or V). The overall flammability profiles of wood substrates coated with the phosphorus-containing compounds were obtained through cone calorimetric measurements. The elemental composition, morphology and chemical natures of the char residues, obtained from the cone tests, were analysed through a variety of spectroscopic, chromatographic and spectrometric means. From the complementary information, obtained through these analyses, some probable mechanistic pathways that underpin the condensed- and gaseous-phase activities of the different additives are suggested. It was found that the inorganic solid additive, i.e., (NH_4_)_2_HPO_4_, underwent a two-step degradation, yielding ammonia gas and phosphoric acid. Furthermore, the liquid additives, owing to their volatility as compared to the solid ones, showed a relatively higher presence in the vapour phase than volatile fragments emanating from the latter ones (i.e., from phosphine and the phosphine oxides).

## 1. Introduction

Wood and its products have been used as a construction material, globally, for thousands of years, primarily owing to their desirable properties like, high strength to weight ratio, ease of availability, durability, etc. Moreover, there is an ample global supply of these materials for the foreseeable future, despite the worldwide trend towards deforestation. Recently, there has been also a renewed interest in the fabrication of products from timber, especially, for their use in high-rise buildings, which can be attributed to its environmentally benign nature and a near carbon neutrality [1]. Furthermore, the use of timber for construction can significantly reduce the environmental impact of construction over the other commonly used construction elements like steel and reinforced concrete [2,3]. However, one important factor which limits the usage of timber as a building material, particularly in larger and high-rise buildings, is its combustible nature [4]. As fire safety is an important criterion of choice for determining the best building material to be used for construction, it is important to address this issue and design strategies to enhance its fire resistance.

Phosphorus-based compounds have shown to be one of the most efficient systems for fire proofing cellulosic materials [5,6]. Generally, phosphorous is incorporated into a polymeric matrix either using an *additive*, or a *reactive*, strategy [7]. The former approach involves the physical incorporation of known fire-retardant (FR) additives into the polymeric substrates, whereas, in the latter technique, phosphorus is chemically incorporated into the macromolecule through chemical modification, or copolymerisation, reactions. As many reactive FR strategies seem to be difficult, or are relatively expensive to implement, additive FR systems are increasingly becoming popular in the commercial sector [7]. In order to effectively design effective strategies to mitigate the ignition propensity of polymeric materials, there is a need to study the mechanisms of flame retardancy that are operating in these substrates as fully as possible. Previous studies have reported that phosphorus flame-retardant mechanism may occur in the condensed (to form phosphoric or polyphosphoric acid species), or in the gaseous phase (for example, in case of low-molecular-weight phosphate esters) and also can vary according to the chemical nature of the phosphorus compound, or the type of polymeric substrate in question [7,8]. The current study attempts to elucidate in detail, the mechanism(s) operating in the condensed- and vapour-phase of some novel formulations using fish gelatin as the base matrix, prepared primarily through an additive approach involving several phosphorus compounds. This was primarily achieved by the analyses of char residues obtained through cone calorimetric tests on the coated wood substrates, and through the identification of the major volatile fragments emanating from the additive themselves. 

## 2. Materials and Methods 

All the additives used in the present study, ((NH_4_)_2_HPO_4_, triphenylphosphine: TPP, triphenylphosphineoxide: TPPO, 9,10-Dihydro-9-oxa-10-phosphaphenanthrene-10-oxide: DOPO, diethylphosphite: DEPi, triethylphosphite: TEPi, triethylphosphate: TEPa, diethylpropylphosphonate: DEPP and diethylbenzylphosphonate: DEBP), except DEBP were purchased from Aldrich Chemical Company, Melbourne, Australia and were used as received. Diethylbenzylphosphonate (DEBP) was synthesized by using the Michaelis–Abruzov reaction [9]. A softwood variety of timber, belonging to the Pine (Pinus radiata, or commonly known as Radiata Pine) family was sourced locally and used as the base substrate.

The char residues for the measurements of phosphorus contents and associated analyses were obtained from the cone calorimetric runs, on virgin and coated timber samples. The formulations for coating the timber substrates were prepared as aqueous colloidal mixtures of fish gelatin (ca. 10 g) and the required amount of additive (i.e., 0.016 mols in all cases) at ca. 60 °C. The different formulations were then uniformly applied onto the top surface of the wood samples (ca. 0.5 mm thickness), and were left to dry in a fume cupboard overnight. The details of sample preparation for the cone tests and test solutions of char residues for inductively-coupled optical emission spectroscopy (ICP-OES) are given elsewhere [10,11]. Here, the measurements of phosphorus contents were done in triplicate by using Shimadzu ICPE-9000 instrument (Shimadzu Scientific Instruments, Melbourne, Australia) and the average values were taken. The details regarding the recording of ^31^P solid-state NMR and Raman spectra are also given in our previous publication [11].

For the GC/MS runs, a Shimadzu GCMS-QP2010 instrument (Shimadzu Scientific Instruments, Melbourne, Australia) with a capillary column with following specifications was used: ZB-5MS; length: 30 m; thickness: 0.5 μm; diameter: 0.25 mm. Dilute solutions of the liquid additive (ca. one or two drops in 1 cm^3^ of methanol were prepared, and were automatically injected (ca. 1 µL) into the port with a split ratio of 10:1. The column oven temperature was set at 45 °C (hold time: 0.5 min) and the injection temperature was set at 250 °C. The temperature ramp was set at 25 °C min^−1^, and final temperature was set at 300 °C (hold time: 1.5 min). The carrier gas pressure was maintained at 86.6 kPa, with a column flow rate of 1.5 cm^3^ min^−1^ where the total flow was set at 154.4 cm^3^ min^−1^. The GC was coupled to the mass spectrometer which employed electron impact ionization. The associated operating parameters of the MS are as follows: ion source temperature: 250 °C, interface temperature: 300 °C; solvent cut time: 3 min; GC program time: 12.2 min; mass range: 40–400 m/z).

Pyrolysis-GC/MS was performed with the pyrolysator Pyroprobe 5000 (CDS Analytical, Inc., Oxford, PA, USA) with platinum filament coupled with gas chromatograph GC7890A (Agilent Technologies, Santa Clara, CA, USA) with GC column HP-5MS (non-polar, length: 30 m; inner diameter: 250 μm; layer thickness: 0.25 μm), (Agilent Technologies, Santa Clara, CA, USA). The carrier gas was helium with a gas flow rate of 1 cm^3^ min^−1^. The GC was equipped with the mass-selective detector MSD 5975C inert XL EI/CI (Agilent Technologies, Santa Clara, CA, USA) with a mass scan range between 15–550 m/z and EI at 70 eV. The samples were pyrolyzed at the temperatures of maximum mass losses found in TGA. The inlet temperature of the GC was variable, the oven temperature program fixed (2 min at 50 °C; heating with 12 K min^−1^ to 280 °C).

## 3. Results and Discussion

The additives chosen for the present study not only differed in terms of the chemical environments but also in terms of the oxidation state of the phosphorus atom (III, or V). They were also constituted of both solids and liquids. Here, the phosphine and phosphine oxides (i.e., TPP, and TPPO and DOPO) can be considered as stable versions of organo-phosphorus compounds with an aromatic structural integrity, whereas the liquid ones (DEPi, TEPi, TEPa, DEPP and DEBP) are relatively easily volatile, and are also more amenable to thermal cracking. The cone calorimetric results clearly indicated that the formulation containing fish gelatin and DOPO exhibited the best fire-proofing effects, as gauged from the values of the relevant parameters. The peak heat release rate (pHRR), total heat released (THR) and effective heats of combustion (EHC) were found to the lowest, as compared to the virgin and other modified substrates [11]. In this context, we have also endeavoured to identify the gaseous phase fragments emanating from these additives, under an electron impact (GC/MS), or upon thermolysis (i.e., pyrolysis-GC/MS)—the details are given in Section 3.2.1 and Section 3.2.2. Whilst some of the additives have been extensively used in the case of synthetic polymers [12,13,14,15,16] to the best of our knowledge, there are only limited information in the literature regarding their use as components for bio-sourced formulations for passively fire protecting ligno-cellulosic materials [10,11].

### 3.1. Condensed-Phase Analysis

#### 3.1.1. Inductively-Coupled Plasma Optical Emission Spectroscopy (ICP-OES)

The phosphorus loadings of the formulations varied within a relatively narrow range that corresponded to a 0.016 molar loading of the additive in each case (i.e., 3.43 to 4.10 wt %), whilst the extents of phosphorus retention in the char residues exhibited a wider range of values (i.e., 3.52 to 7.82 wt %). Therefore, it can be inferred that the phosphorus-containing additives do exhibit varying degrees of condensed phase activity (see Table 1). However, the exact extents of this effect cannot be directly gauged from the values of the initial phosphorus loading, or retention, owing to the fact that the char residue can be very inhomogeneous and the exact contributions of the underlying burnt/partially burnt wood towards the residue, therefore, cannot be determined with any certainty.

The initial P loading for the various formulations used for cone calorimetric tests can be calculated, as given below. Furthermore, from the char yields obtained through the cone runs and extent of phosphorus retention in the char residues, the wt % of phosphorus liberated into the vapour phase can be also calculated, as shown below (see also in Table 1).

(1) $$\mathrm{Wt}\;\%\;\mathrm{of}\;P=\frac{\frac{31}{\mathrm{Fr}.\;\mathrm{mass}\;\mathrm{of}\;\mathrm{additive}}\times \mathrm{mass}\;\mathrm{of}\;\mathrm{additive}\times 100}{\left(\mathrm{mass}\;\mathrm{of}\;\mathrm{fish}\;\mathrm{gelatin}+\mathrm{mass}\;\mathrm{of}\;\mathrm{additive}\right)}$$

For example, fish gelatin when mixed with DOPO (Fr. mass = 216)

(2) $$\mathrm{Wt}\;\%\;\mathrm{of}\;P=\frac{\frac{31}{216}\times 3.46\times 100}{\left(10+3.46\right)}=3.68$$

(3) $$P\;\mathrm{loading}\;\left(\mathrm{theoretical}\;\max.\;\mathrm{in}\;\mathrm{the}\;\mathrm{char}\right)=\frac{P\;\mathrm{loading}\;\left(\mathrm{initial}\right)}{\mathrm{Char}\;\mathrm{yield}\;\left(\mathrm{from}\;\mathrm{cone}\right)}\times 100$$

(4) $$P\;\mathrm{loading}\;\left(\mathrm{theoretical}\;\max.\;\mathrm{in}\;\mathrm{the}\;\mathrm{char}\right)=\frac{3.68}{20}\times 100=16$$

As expected, the liquid additives, owing to their volatility as compared to the solid ones, showed a relatively higher presence in the vapour phase. Furthermore, it can be seen that relatively substantial amounts of phosphorus-containing species from phosphine and the phosphine oxides were also presumably released into the vapour phase through fragments emanating from the thermal pyrolysis in the condensed phase [5]. The chemical environment of the phosphorus moieties retained in the condensed phase can be assessed from the ^31^P solid-state NMR (Section 3.1.2 below) [12] and the fragmentation patterns of the additives can be deduced from GC/MS, or pyrolysis-GC/MS (Section 3.2.1 and Section 3.2.2, respectively) [17].

#### 3.1.2. Solid-State NMR Spectra

In the following Figure 1, Figure 2, Figure 3, Figure 4, Figure 5, Figure 6, Figure 7, Figure 8 and Figure 9, the ^31^P signals obtained through solid state NMR measurements on powdered char residues collected from the cone experiments are given (see also Table 2):

As expected, phosphorus acid species (∂ ~ 0 ppm), or oligomeric phosphate moieties (∂ ~ −10 ppm), are generated from the initial cracking/condensation reactions of AP, DEPi, TEPi, TEPa, DEPP and DEBP [12]. However, cracking of TPP, TPPO and DOPO are bound to produce phenyl, or biphenyl species, and the phosphorus atom is likely to go into vapour phase either in the elemental form and/or as oxide species [16]. Therefore, the signals in the latter cases can be attributed to unburnt phosphine (TPP), or phosphines oxide (TPPO, or DOPO), where the coupling patterns (scalar, including long-range) owing to ^1^H-^31^P are also evident. The features of such patterns in a solid-state NMR spectrum (i.e., due to any unburnt additive) are likely to be often complicated by the presence of rather prominent spinning side bands (see for example, in case of TPP, TPPO and DEPi). By looking at the complementary information that was gathered through pyrolysis-GC/MS and GC/MS, we were able to formulate some plausible fragmentation pathways in the case of the additives (see in Section 3.2).

#### 3.1.3. Raman Spectroscopy

The structural features of char residues can be deduced from observing the relevant signal intensities in the Raman spectra [11,18,19]. In the present study, we recorded the Raman spectra from representative char residues where the intensities of the graphitic (*G* band, associated with the vibrations of plane terminal glass carbons’ unorganized carbon structure) and amorphous (*D* band, owing to an *E*_2*g*_ vibrational mode of graphitic *sp*^2^ bonded carbon atoms’ graphitic layered structure) were compared [11]. Hence, the ratios of the intensities of the bands (i.e., *I_G_*/*I_D_*), as measured through their respective areas, were different for all the test samples, and were indicative of the degree of graphitization of the carbonaceous residues obtained upon burning (Table 3). Generally, higher intensity of the *G* band with respect to the *D* band points to a more coherent char structure. The char residue from fish gelatin + TPPO was evidently richer in the graphitic region (ratio = 1.32) as compared to the residues of other formulations, and the corresponding value was the least in the case of fish gelatin + DEPP (ratio = 0.33). In the case of other systems, only fish gelatin + DEBP showed a higher value (ratio = 0.93), as compared with the unmodified substrate (0.73), whereas all other systems generally showed lower values.

### 3.2. Vapour-Phase Analyses of the Additives (i.e., Hyphenated Techniques)

With a view to obtaining more insight into the thermal cracking of the additives, we carried out some gaseous phase analyses of the volatiles emanating from them using ‘hyphenated’ techniques, such as GC-MS (for the liquid additives: DEPi, TEPi, TEPa, DEPP and DEBP), or pyrolysis-GC/MS (for solid additives: TPP, TPPO and DOPO). Here it should be noted that thermolysis of these additive under a flaming mode (i.e., under relatively higher heating rates and within lower time scales, and with varying degrees of oxygen ingression) cannot be directly compared to those occurring under a progressive heating according to a predetermined temperature ramp, followed by an electron impact (as in a GC/MS), or under a thermally induced degradation at a preset temperature (in pyrolysis-GC/MS). Albeit, the basic assumption that regardless of the mode and rate of inducing bond cleavages in the additives, the weaker bonds are broken to result in relatively stable species can be considered as valid within reason.

The details of the liquid samples, retention times and major species recorded by the mass spectrometer are given in table below (Table 4). In the case of the liquid samples that underwent fragmentation, the molecular ion peak was only shown up in the case of TEPa, where less conspicuous signals corresponding to [M ± 1]^+^ were evident in the cases of DEPi and TEPa. In the case of the phosphonate additives, such as DEPP and DEBP, the molecular ions initially formed seem to have undergone rapid fragmentation resulting in stable species (with m/z values of 125 and 95 respectively).

#### 3.2.1. Gas Chromatography-Mass Spectrometry (GC/MS)

From the presence of the [M]^+^, or [M]^+^ ± 1, and/or nature of [M]^+^ (100%) and the major species, we could formulate the following fragmentation pathways in the case of the liquid additives, as follows (see in Figure 10, Figure 11, Figure 12, Figure 13 and Figure 14):

#### 3.2.2. Pyrolysis-Gas Chromatography/Mass Spectrometry (Pyrolysis-GC/MS)

The temperatures for pyrolysis of the solid organophosphorus compounds were selected on the basis of the first derivatives of their thermograms, in nitrogen at heating rate of 10 °C min^−1^ and from 30 to 900 °C, as follows: TPP = 288 °C; TPPO = 340 °C; DOPO = 381 °C. The inorganic solid additive, i.e., (NH_4_)_2_HPO_4_, was not included in this study as it is assumed to undergo a two-step degradation yielding ammonia gas and phosphoric acid in the first step (ca. 70 °C), followed by the degradation of the product, ammonium dihydrogen phosphate to produce oxides of phosphorus and nitrogen and ammonia (ca. 155 °C) degradation [20].

It is very clear from the GC/MS of TPP and TPPO that the molecular ion initially produced showed typical fragmentation pattern of aromatic phosphine and phosphine oxide (above their normal melting points, but below the corresponding boiling points), as the case may be. However, in the case of DOPO, three distinct peaks were obtained in the chromatogram, and the corresponding mass spectra were indicative of DOPO (retention time = 18.83 min); degradation product of DOPO (retention time = 15.7 min, with fragments from DOPO with m/z values of: 152, 170, 199 and 200); *o*-hydroxybiphenyl (retention time = 13.1 min)—see details in Table 5 below:

Judging primarily from the results of the pyrolysis-GS/MS studies of the solid organophosphorus additives (TPP, TPPO and DOPO), and the corresponding outcomes from GC/MS for the liquid additives, we envisage the following degradation pathways of these compounds (Figure 15 and Figure 16).

**Figure 1:**
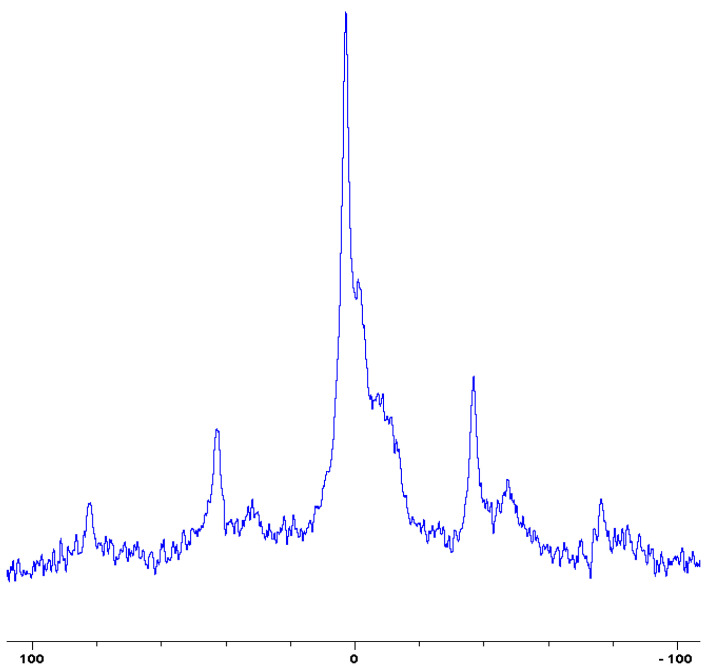
Solid-state ^31^P-NMR spectrum of char obtained from fish gelatin + triphenylphosphine (TPP) (the abscissa denotes the chemical shift values, *δ*, in ppm and the ordinate corresponds to the signal intensity in arbitrary units).

**Figure 2:**
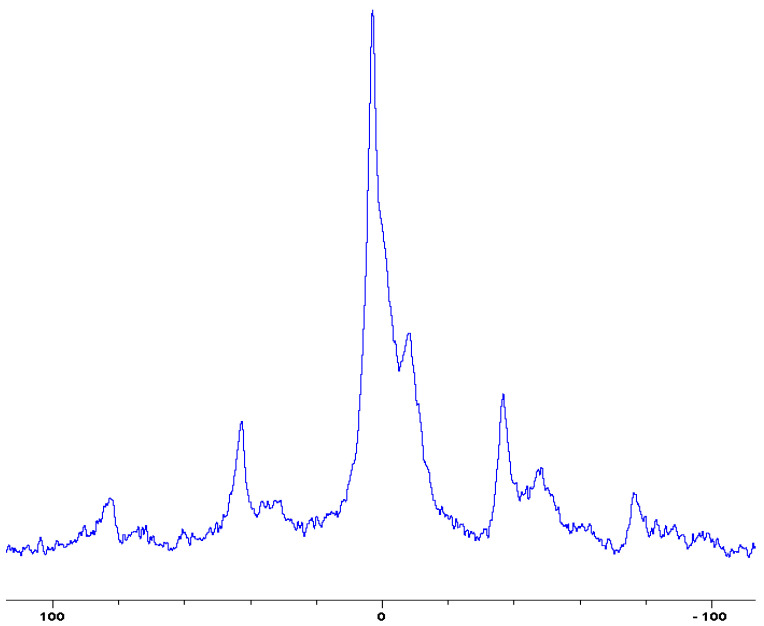
Solid-state ^31^P-NMR spectrum of char obtained from fish gelatin + triphenylphosphineoxide (TPPO) (the abscissa denotes the chemical shift values, *δ*, in ppm and the ordinate corresponds to the signal intensity in arbitrary units).

**Figure 3:**
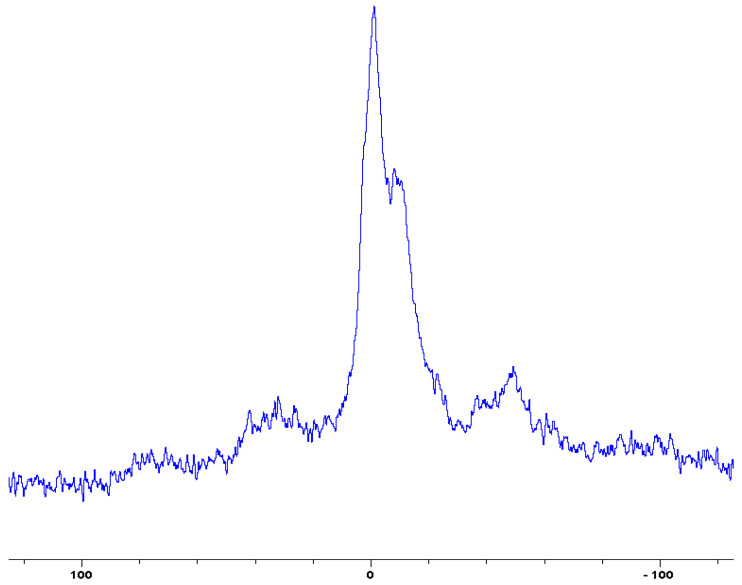
Solid-state ^31^P-NMR spectrum of char obtained from fish gelatin + 9,10-Dihydro-9-oxa-10-phosphaphenanthrene-10-oxide (DOPO) (the abscissa denotes the chemical shift values, *δ*, in ppm and the ordinate corresponds to the signal intensity in arbitrary units).

**Figure 4:**
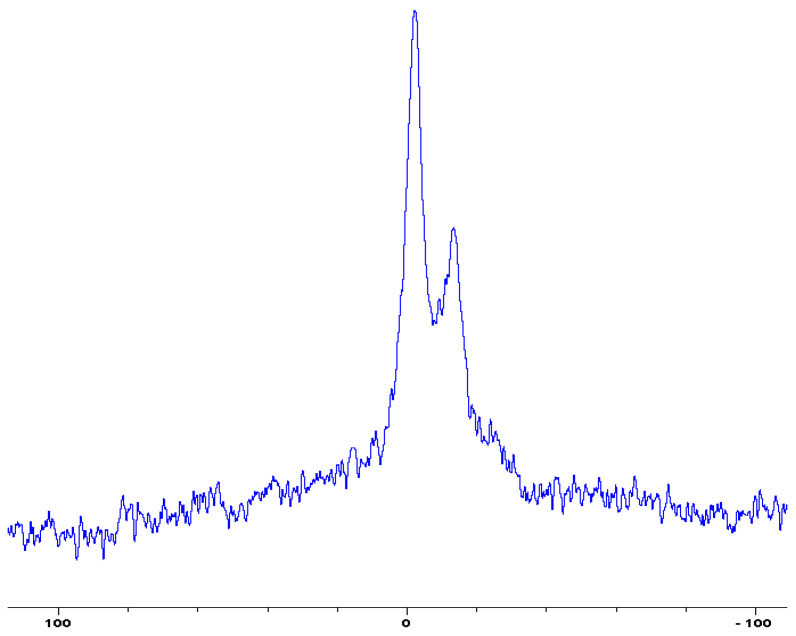
Solid-state ^31^P-NMR spectrum of char obtained from fish gelatin + (NH_4_)_2_HPO_4_ (the abscissa denotes the chemical shift values, *δ*, in ppm and the ordinate corresponds to the signal intensity in arbitrary units).

**Figure 5:**
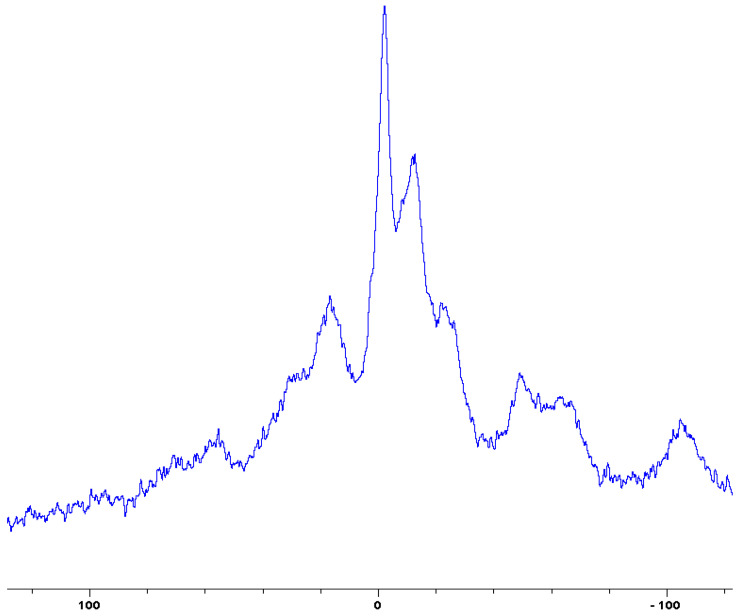
Solid-state ^31^P-NMR spectrum of char obtained from fish gelatin + diethylphosphite (DEPi) (the abscissa denotes the chemical shift values, *δ*, in ppm and the ordinate corresponds to the signal intensity in arbitrary units).

**Figure 6:**
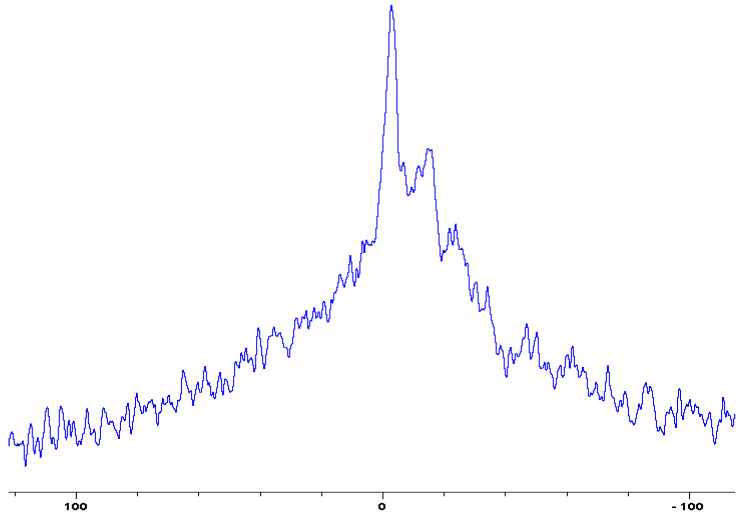
Solid-state ^31^P-NMR spectrum of char obtained from fish gelatin + triethylphosphite (TEPi) (the abscissa denotes the chemical shift values, *δ*, in ppm and the ordinate corresponds to the signal intensity in arbitrary units).

**Figure 7:**
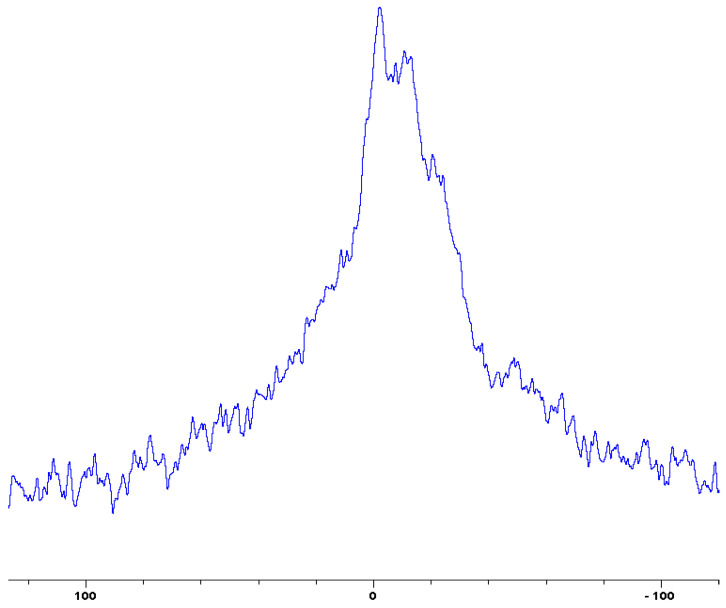
Solid-state ^31^P-NMR spectrum of char obtained from fish gelatin + triethylphosphate (TEPa) (the abscissa denotes the chemical shift values, *δ*, in ppm and the ordinate corresponds to the signal intensity in arbitrary units).

**Figure 8:**
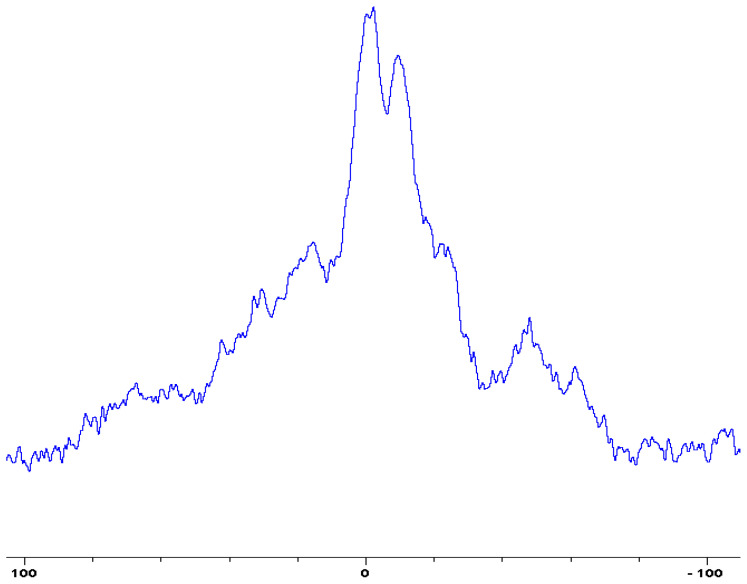
Solid-state ^31^P-NMR spectrum of char obtained from fish gelatin + diethylpropylphosphonate (DEPP) (the abscissa denotes the chemical shift values, *δ*, in ppm and the ordinate corresponds to the signal intensity in arbitrary units).

**Figure 9:**
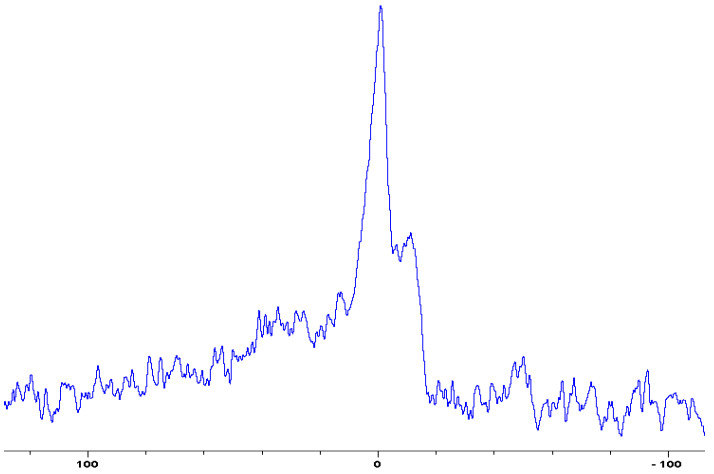
Solid-state ^31^P-NMR spectrum of char obtained from fish gelatin + diethylbenzylphosphonate (DEBP) (the abscissa denotes the chemical shift values, *δ*, in ppm and the ordinate corresponds to the signal intensity in arbitrary units).

**Figure 10:**
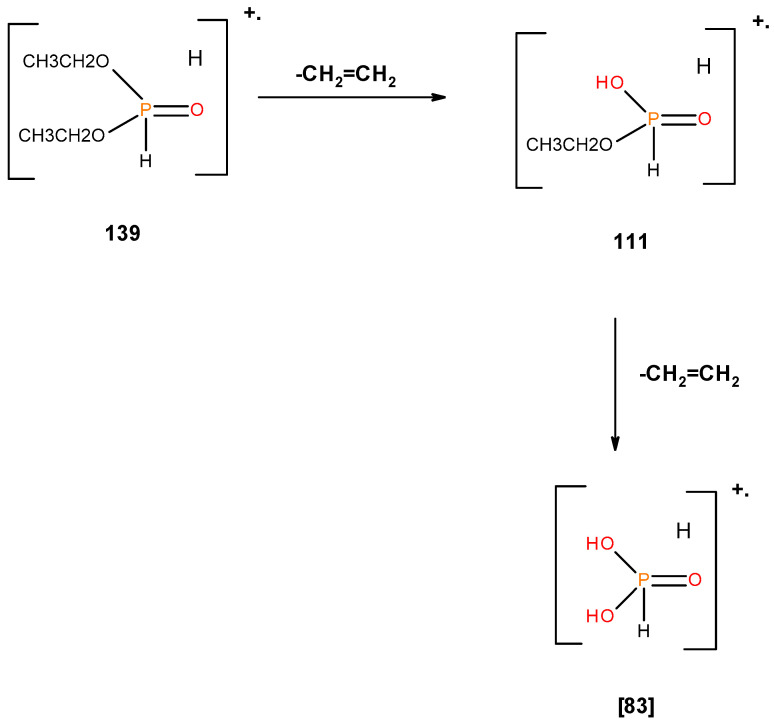
Possible fragmentation pattern for DEPi.

**Figure 11:**
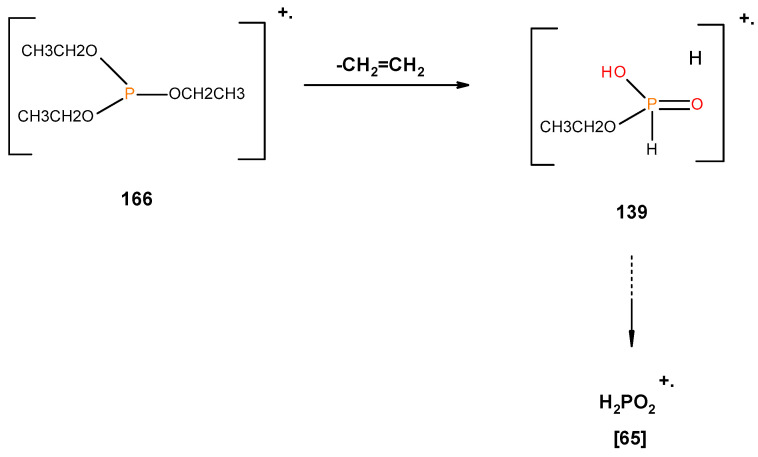
Possible fragmentation pattern for TEPi.

**Figure 12:**
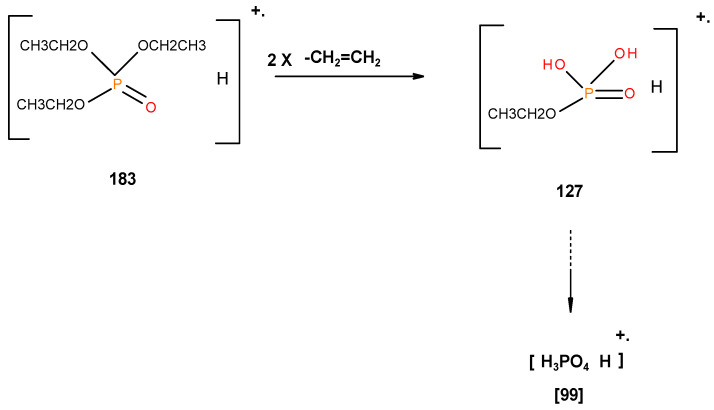
Possible fragmentation pattern for TEPa.

**Figure 13:**
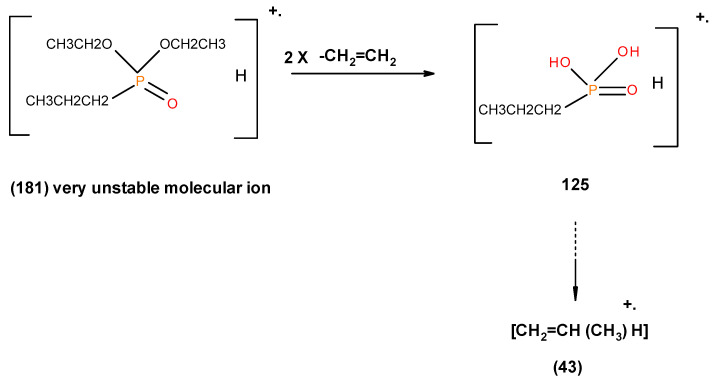
Possible fragmentation pattern of DEPP.

**Figure 14:**
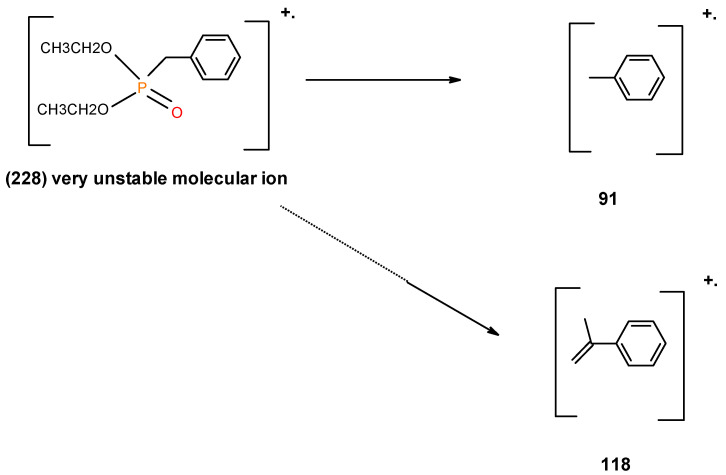
Possible fragmentation pattern of DEBP.

**Figure 15:**
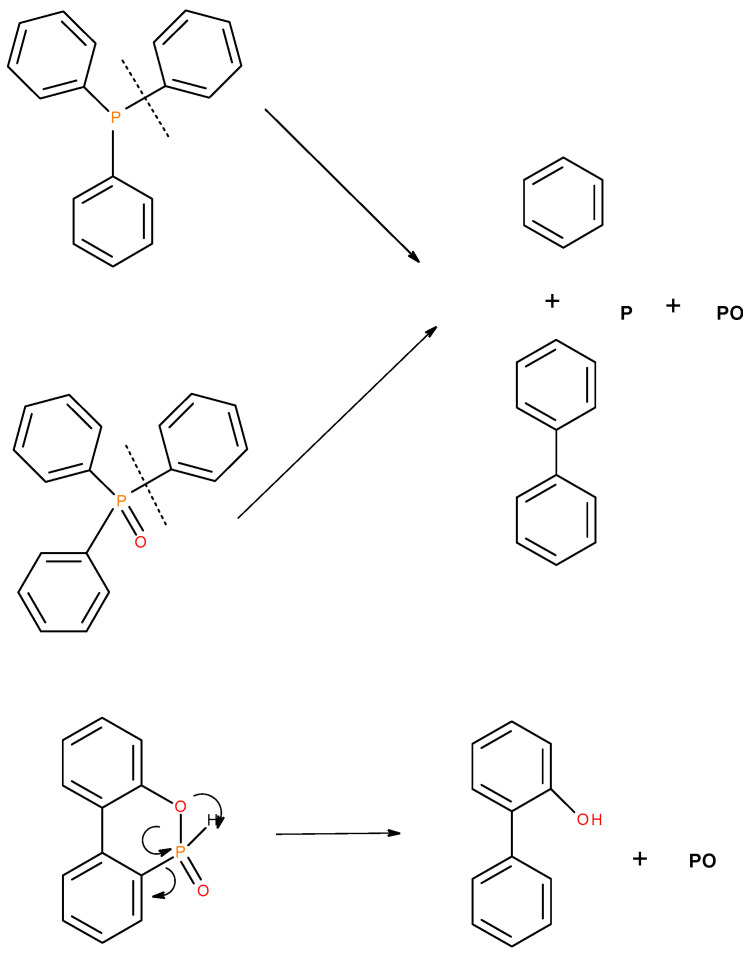
A schematic diagram of production of phosphorus-centred species in the gaseous phase in the case of solid organophosphorus additives.

In the case of the solid additives such as TPP and TPPO, typically above their melting points but below their boiling temperatures, volatilization is first effected followed by the cleavage of the P-Ar bonds to form aromatics, either phenyl or biphenyl compounds, and resulting in the release of P, oxygenated phosphorous moieties in the gaseous phase. Elemental phosphorus and/or oxides are found to be active in the vapour phase [7]. Here it should be noted that the presence of phosphorus (as gauged through ICP/OES: Table 1) and the chemical environment of phosphorus bearing moieties in the condensed phase (see the respective ^31^P NMR spectrum: in Figure 1, Figure 2, Figure 3, Figure 4, Figure 5, Figure 6, Figure 7, Figure 8 and Figure 9) clearly indicate that the phosphine, or phosphine oxide as the case may be, are retained as such without undergoing thermal degradation.

**Figure 16:**
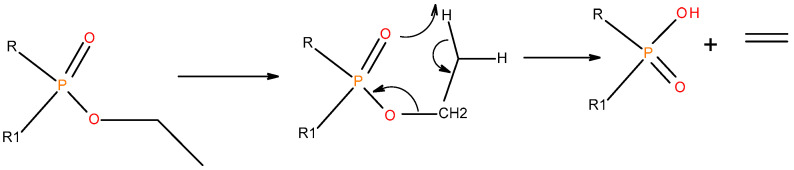
A general schematic diagram of production of phosphorus acidic species, in the condensed phase through the elimination of ethene from the ester side arms of the liquid additive.

In the case of the liquid additives, such as DEPi, TEPi, TEPa, DEPP and DEBP, it appears that the thermal cracking of the side phosphate, or phosphonate, group is favoured through a preferred cyclic intermediate liberating ethene, and the latter can be considered as entropically favourable—see Figure 16 [7]. Furthermore for the liquid additives, especially, with the P-atom in the oxidation state of V, elimination can be considered as very feasible through the six-membered ring intermediate, where as in the case of the phosphite (where the P-atom is in the oxidation state of III) it has to go through a five-membered ring intermediate, or through a six-membered analogue if prior oxidation of P(III) to P(V) is assumed [7,12,21]. It is relevant to note here that there is unequivocal evidence for the presence of phosphorus acid species in the char residues from the corresponding ^31^P solid-state NMR (signals centred at ∂ ~ 0.0 ppm).

## 4. Conclusions

Through the present work we have endeavoured to decipher the degree, nature and relative predominance of the condensed- and gaseous-phase activities of the different additives through a combination of standard (atomic absorption, solid-state NMR and Raman spectroscopies), and optionally through ‘hyphenated’ (pyrolysis-GC/MS and GC/MS), techniques. It is to be noted here that the loading of the various phosphorus containing additives was normalized in terms of their molar content (0.016 mols). This in turn has resulted in varying amounts of P-loadings (in wt %). Therefore, no meaningful correlation(s) between the flame retardant performance and the chemical environments and oxidation state of the P atom can be drawn. The influence of the oxidation state, and hence possible variation of the chemical environment, of the P atom in fire retardant formulations is reported elsewhere [1,21,22,23,24,25]. From the collated empirical data, the following conclusions can be drawn:

- The inorganic solid additive, i.e., (NH_4_)_2_HPO_4_, underwent a two-step degradation, yielding ammonia gas and phosphoric acid in the first step, followed by the degradation of the product, ammonium dihydrogen phosphate to produce oxides of phosphorus and nitrogen and ammonia;
- As expected, the liquid additives, owing to their volatility as compared to the solid ones, showed a higher presence in the vapour phase. Furthermore, it can be seen that relatively substantial amounts of phosphorus-containing species from phosphine and the phosphine oxides were also presumably released into the vapour phase through fragments emanating from the thermal pyrolysis in the condensed phase
- The ^31^P-NMR spectra showed the ‘phosphorus’ acid species (∂ ~ 0 ppm), or oligomeric phosphate moieties (∂ ~ −10 ppm), are generated from the initial cracking/condensation reactions of AP, DEPi, TEPi, TEPa, DEPP and DEBP. However, cracking of TPP, TPPO and DOPO are bound to produce phenyl, or biphenyl species, and the phosphorus atom is likely to go into vapour phase either in the elemental form and/or as oxide species;
- The Raman spectra exhibited different degrees of graphitization, as revealed by the varying ratios of the areas corresponding to ordered and amorphous domains;
- From the complementary information that was gathered though vapour analyses, some probable mechanistic pathways that underpin the condensed- and gaseous-phase activities of the different additives were suggested
- In summary, it can be inferred that the organic solid additives (TPP, TPPO and DOPO) are predominantly active in the vapour phase, whereas the liquid additives (DEPi, TEPi, TEPa, DEPP and DEBP) can considered as more active in the condensed phase. The inorganic additive, AP, can assumed to be more active in the condense phase just the liquid additives.

## Figures and Tables

**Table 1:** Phosphorus contents, determined through inductively-coupled optical emission spectroscopy (ICP-OES) measurement, in the coating formulations and corresponding char residues.

Sl. No.	Sample	wt % P in the Coating	Char Yield (wt %) from Cone	Theoretical Maximum of P Loading in the Char (wt %)	P Loading (ppm)	wt % P in the Char	wt % of P in the Gaseous Phase
1	Fish gelatin + AP	4.10	21	19.52	36.6	7.82	59.93
2	Fish gelatin + DOPO	3.68	23	16.00	24.45	6.06	62.12
3	Fish gelatin + TPP	3.48	20	17.40	14.73	4.46	74.36
4	Fish gelatin + TPPO	3.43	* ---	* ---	11.23	3.50	* ---
5	Fish gelatin + DEPi	4.05	20	20.25	9.02	4.68	76.89
6	Fish gelatin + TEPi	3.97	21	18.90	3.84	2.83	85.02
7	Fish gelatin + TEPa	3.62	21	17.24	2.15	3.16	81.67
8	Fish gelatin + DEPP	3.85	19	20.26	4.63	2.04	89.93
9	Fish gelatin + DEBP	3.63	20	18.15	1.68	1.85	89.80

* could not be determined as the cone instrument erroneous values for the char yield.

**Table 2:** Chemical shift values (*∂* in ppm) of P nucleus in the additives and in the char residue.

Sl. No.	Additive	∂ Value for ^31^P of the Additives * (Solution-State)	∂ Value for ^31^P of the Char Residue ^#^
1	AP	0.02	−2.3 (−13.4)
2	TPP	−7.10	2.9 (−8.5)
3	TPPO	27.6	2.9 (−8.5)
4	DOPO	16.7	−1.3 (−9.8)
5	DEPi	7.30	−2.3 (−13.1)
6	TEPi	7.30	−3.3 (−15.0)
7	TEPa	−1.00	−2.6 (−10.8)
8	DEPP	32.2	−1.3 (−9.1)
9	DEBP	26.4	−1.0 (−10.8)

* from ^31^P solution-state NMR spectrum—here the protons were decoupled during the acquisition of ^31^P NMR, and hence do not show ^1^H-^31^P coupling patterns; ^#^ the major peak from solid-state NMR of the char residue, where the values in the parentheses represent the chemical shift (∂) of the ‘shoulder’ peak. It is relevant to note here that the ^31^P solid state NMR of the char residues were acquired without broad band decoupling of residual protons that might be present in the char residues.

**Table 3:** Details of Raman spectra of char residue.

Sl. No.	Char Residue	Intensity of the G Band (*I_G_*)	Intensity of the D Band (*I_D_*)	Ratio (*I_G_*/*I_D_*)
1	Unprotected wood	18,030	24,830	0.73
2	Fish gelatin + AP	21,850	30,360	0.72
3	Fish gelatin + TPP	476,979	1,099,132	0.43
4	Fish gelatin + TPPO	450,413	343,278	1.32
5	Fish gelatin + DOPO	648,452	1,030,772	0.63
6	Fish gelatin + DEPi	580,753	1,002,797	0.58
7	Fish gelatin + TEPi	515,705	954,779	0.54
8	Fish gelatin + TEPa	570,280	943,631	0.60
9	Fish gelatin + DEPP	402,813	1,238,529	0.33
10	Fish gelatin + DEBP	846,410	913,333	0.93

**Table 4:** Retention times and fragmentation features of the various liquid additives.

Sample	Chemical Structures	Retention Time (min)	Molar Mass	[M]^+.^	[M ± 1]^+.^	[M] (100%)	Other Predominant Species/Remarks
DEPi	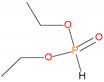	4.7	138	---	139	83	111
TEPi	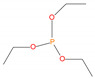	3.9	166	166	---	65	139
TEPa	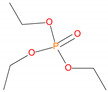	5.2	182	---	183	99	127
DEPP	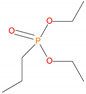	5.0	180	---	---	125	43
DEBP	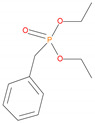	8.0	228	---	---	91	118

**Table 5:** Retention times and fragmentation features of the various solid additives.

Sample (Chemical Name)	Chemical Structures	mp/bp (°C)	Pyrolysis Temp. (°C)	Retention Time (min)	Molar Mass	[M]^+^ ± 1
TPP	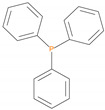	80/377	288	18.8	263	262
TPPO	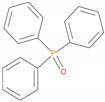	154–158/360	340	21.4	278	277
DOPO	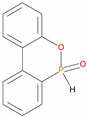	116–120/200 (at 1mm Hg)	381	18.8 * 15.7/ ^#^ 13.1	216	216

* indicative of degradation product of DOPO; ^#^ indicative of *o*-hydroxybiphenyl.
